# First molecular-based detection study of *Leishmania infantum* in the Tapirapé indigenous population in the Brazilian Amazon

**DOI:** 10.1590/1414-431X2021e11654

**Published:** 2022-02-04

**Authors:** D.S.C. Freitas, R.E. Silva, J.O.J. Costa, D.V. Markus, H.S. Soares, A.H.H. Minervino, J.T.R. Lima, S.M. Gennari, A. Marcili

**Affiliations:** 1 Faculdade de Medicina, Universidade de Santo Amaro, São Paulo, SP, Brasil; 2 Programa de Pós-Graduação em Medicina Animal, Bem Estar e Saúde, Universidade de Santo Amaro, São Paulo, SP, Brasil; 3 Laboratório de Doenças Parasitárias, Departamento de Medicina Veterinária Preventiva e Saúde Animal, Faculdade de Medicina Veterinária e Zootecnia, Universidade de São Paulo, São Paulo, SP, Brasil; 4 Laboratório de Sanidade Animal, Universidade Federal do Oeste Pará, Santarém, PA, Brasil

**Keywords:** Phlebotominae, Confresa, Tapi'itãwa, PCR, Amazonia

## Abstract

Species of the genus *Leishmania* parasitize mammals and have life cycles that alternate between vertebrate and invertebrate hosts. Most species develop in a hematophagous arthropod and infect a specific vertebrate host that may belong to diverse orders and families. Visceral leishmaniasis is a chronic zoonosis with a wide geographic distribution, affecting 350 million people globally, mostly in areas with a high risk of infection. In Brazil, this disease not only has a high incidence but is also expanding to new areas, both in urban centers and rural areas, including territories with tribal communities, due to increasing human intervention. The objective of this study was to perform cathepsin L-*like* gene-based molecular diagnosis of *Leishmania infantum* in the indigenous Tapirapé ethnic group in the state of Mato Grosso. From the 372 individuals assessed, only 0.8% (3/372) tested positive for *L. infantum*, all from the same village (Urubu Branco). Despite the small number of infected individuals, this study demonstrates the first human cases of *Leishmania infantum* infection in this population, suggesting the need for regular monitoring of visceral leishmaniasis in the area and leading to a broad discussion on the planning and implementation of public health measures for the indigenous population, while respecting their distinctive territories and culture.

## Introduction

Visceral leishmaniasis is classified as a zoonosis and is a neglected tropical disease with an estimated incidence of 50,000 to 90,000 new cases per year according to the World Health Organization (WHO). More than 90% of the cases are concentrated in only ten countries worldwide, including Ethiopia, Eritrea, India, Iraq, Kenya, Nepal, Somalia, South Sudan, Sudan, and Brazil (1,2).

Leishmaniasis is caused by *Leishmania infantum* (3,4), and the transmission cycle involves a sand fly vector belonging to the *Lutzomyia* genus of Phlebotominae subfamily (5) and a variety of vertebrate hosts. The domestic dog is the main reservoir, and wild mammals such as marsupials, bats, horses, and felines are hosts of secondary epidemiological importance (6-11).

In this multifactorial epidemiological context, the disease is prevalent throughout Brazil, affecting many states and different biomes (12). In absolute numbers, the northeast region of Brazil has the highest concentration of cases, but attention must be paid to frequently expanding areas with new cases, which bring transmission circuits closer to urban areas (13,14).

The rapid advance of the disease, however, is not restricted to large urban centers and metropolitan areas. The same occurs in the countryside, where anthropic interventions are mainly related to agriculture, cattle ranching activities, and mining, causing drastic changes in the landscape and topography (15,16).

In recent years, amidst these various epidemiological elements involved in the understanding of visceral leishmaniasis, it has become apparent that the “indigenous population” comprises an extremely neglected portion of the population in Brazil. Indigenous health care was planned with the creation of the National Policy for Indigenous Health Care in 1999, in conjunction with the provisions of Law 9.836, of September 23, 1999, which adopted a biomedical-scientific model of health promotion. The organizational chart of this support network foresees that villages provide basic care, whereas medium and high complexity care is directed to and supported by the Indigenous Health Care Homes (CASAI). However, there is a discontinuity of these services because of multiple factors, notably language and cultural differences, geographical barriers, acceptance of professional services by only a proportion of the natives, and problems with the financial maintenance of this network. Furthermore, the discontinuity of public policies aimed at indigenous populations results in high rates of morbidity and mortality, especially with regard to infectious and parasitic diseases (17-19).

The constant and increasing anthropic interference in indigenous territories, together with a series of interruptions in epidemiological investigations resulting from discontinuity in planning, implementation, and effectiveness of public policies on primary health care that respect the cultural and logistical issues of these populations, ultimately increase the chances of epidemic outbreaks, as has been observed with other infectious diseases (20-24).

The aim of this study was to describe the prevalence of *Leishmania infantum* infections in the indigenous population of Tapirapé, in the Brazilian Amazon, in order to obtain new epidemiological information.

## Material and Methods

The study was conducted after approval by the Research Ethics Committee of the National Indian Foundation (FUNAI; protocol number 08620.002433/2007-990).

The Urubu Branco indigenous land, inhabited by the Tapirapé people of the Tupi-Guarani linguistic family, was selected for this study. This reserve of 168,000 hectares is located in an area of the legal Amazon that includes seven villages: Urubu Branco (10°40'16.1''S, 51°21'13.8''W), Sapeva (10°48'12.2''S, 51°17'12.8''W), Córrego da Onça (10°42'27.5''S, 51°18'49.0''W), Buriti II (10°39'33.6''S, 51°17'41.2''W), Codebra (10°52'09.6''S, 51°15'37.8''W), Xexéu (10°51'20.6''S, 51°18'19.8''W), and Santa Laura (10°36'23.9''S, 51°10'47.9''W) distributed in the municipalities of Confresa, Porto Alegre do Norte, and Santa Terezinha in the northeast of Mato Grosso state in the central-west region of Brazil.

Blood samples were collected from volunteers in the reserve who were over three years of age. The samples included 212, 63, 44, 32, 17, and 4 individuals from Urubu Branco, Santa Laura, Córrego da Onça, Sapeva, Codebra, and Buriti II, respectively, totaling 372 individuals.

Whole-blood samples were collected using EDTA as an anticoagulant in vacuum tubes, stored as frozen sub-aliquots in plastic microtubes at -20°C, and sent to the laboratory. DNA was extracted from the blood samples using the commercial PureLink^®^ Genomic DNA Mini Kit (Thermo Fisher, USA), followed by spectrophotometric quantification to verify the extraction efficiency.

For specific molecular diagnosis of *Leishmania infantum*, PCR was performed using cathepsin L-*like* gene (7).

## Results

Of the 372 indigenous individuals tested, the molecular diagnosis revealed three positive samples, corresponding to a percentage of 0.8% (3/372). Two positive samples were from females and one from a male, aged between 17 and 37 years, all residents of Urubu Branco.

## Discussion

This study reports for the first time infections by *Leishmania infantum* in the Tapirapé indigenous population assessed by molecular tests, confirming the circulation of *L. infantum* in this population. A previous study indicated that the occurrence of antibodies anti-*Leishmania* in the canine population residing in these villages was greater than 8%, but in humans, only two samples presented reactive results to *L. amazonensis* (25). The different serological tests used may vary in sensitivity and specificity, depending on the method (26). Importantly, the higher percentage in dogs and human reaction to *L. amazonensis* may be due to discrepancies in these serological tests, such as specificity failures that induce serum cross-reactivity phenomena (27,28) and the molecular diagnosis based on cathepsin L-*like* being specific to *L. infantum* (7).

Methodology, type of antigen used, time of infection, and host characteristics directly influence the performance of serological testes (26). Thus, the use of molecular tools with high sensitivity and specificity are more effective in assisting the development of public health policies.

Entomological surveys conducted in indigenous villages in the state of Mato Grosso show the presence of species of sand flies that are important vectors of *Leishmania infantum*, notably *Lutzomyia longipalpis* and *Lutzomyia cruzi* (29), suggesting that these species are also present around Urubu Branco Indigenous Land. Among the villages studied, this is the one with the highest population density and has also experienced many anthropic interferences, resulting in many ecological tensions that favor these vector species, which explains all the epidemiological elements necessary to maintain the transmission chain identified in this study.

Various authors suggest that the increasing occurrence of these vector species in indigenous areas is the result of social tensions faced by these populations, triggered by economic activities that threaten the integrity of their territories, fragment natural space, and disrupt systems of ethnic and social organization, resulting in migration and displacement (29,30). These episodes of fragmentation are of particular concern because they allow the expansion of areas of direct interaction between forest ecotopes and peri-domiciles of low health security, in addition to providing pathways for the dispersion of positive dogs between different population centers.

Another element that may explain the presence of sand flies and the resulting transmission of the disease in these peri-domicile areas is the increase in the population of dogs kept either for affection reasons or subsistence hunting. In addition, extractivism and the presence of livestock, including chickens and pigs, which, although not actual hosts of the parasite, serve as blood-meal sources for insect vectors (30,31).

This study suggests that specific molecular tests for *Leishmania infantum* may be an alternative to the serological methods commonly used and recommended by health agencies, as they are important tools for solving problems of sensitivity and specificity of serological tests (25). Therefore, this molecular diagnostic test can be efficiently used in routine diagnosis of infections in patients and in epidemiological surveys involving humans, animal hosts, and insect vectors (7).

These results, together with previous epidemiological studies on visceral leishmaniasis in indigenous populations, highlight the social vulnerabilities faced by these populations when they are exposed and issues related to marginalization of access to basic health care systems, which leads to reduced testing for chronic infectious diseases such as visceral leishmaniasis (32). Another factor that deserves particular attention is the demand for access to the formal education system, as studies suggest that schooling acts as a protective factor for various chronic infectious diseases, confirming the potential of schools as decentralized platforms for strengthening the concept of health education (33).

Additionally, it is important to consider the occurrence of infectious comorbidities such as HIV, influenza viruses, and other systemic parasites in these populations, as these diseases can lead to a direct increase in the probability of occurrence and spread of visceral leishmaniasis. Effective management of these issues require health policies that consider multiple interventions in an integrated manner with contextualized initiatives that go beyond conventional medical curative prescription-based approaches (34-38).

These epidemiological parameters are affected by multiple factors, including a rapid increase in the rate of deforestation in the legal Amazon, weakening and dismantling of public policies related to sustainable indigenous development, the exacerbation of conflicts related to the demarcation of Federal indigenous reserves, and a mistaken Federal vision that opposes the protection of natural resources to economic development (39,40).

In conclusion, a small number of infected individuals were found in the studied area, but as these were the first recorded human cases of *Leishmania infantum* infection in this locality we can draw important conclusions: 1) there is a need to adopt consistent and effective measures for monitoring visceral leishmaniasis in this area, and 2) in broader terms, there is a need to plan and implement public health policies that are appropriate to the logistical characteristics and respect the cultural components of these indigenous peoples who have inhabited these territories for centuries and thus have legitimate rights to them.
